# Combatting the Increasing Threat of Vector-Borne Disease in the United States with a National Vector-Borne Disease Prevention and Control System

**DOI:** 10.4269/ajtmh.18-0841

**Published:** 2018-11-29

**Authors:** Lyle R. Petersen, Charles B. Beard, Susanna N. Visser

**Affiliations:** Division of Vector-Borne Diseases, National Center for Emerging and Zoonotic Infectious Diseases, Centers for Disease Control and Prevention, Fort Collins, Colorado

## Abstract

Reported cases of vector-borne diseases in the United States have more than tripled since 2004, characterized by steadily increasing incidence of tick-borne diseases and sporadic outbreaks of domestic and invasive mosquito-borne diseases. An effective public health response to these trends relies on public health surveillance and laboratory systems, proven prevention and mitigation measures, scalable capacity to implement these measures, sensitive and specific diagnostics, and effective therapeutics. However, significant obstacles hinder successful implementation of these public health strategies. The recent emergence of *Haemaphysalis longicornis*, the first invasive tick to emerge in the United States in approximately 80 years, serves as the most recent example of the need for a coordinated public health response. Addressing the dual needs for innovation and discovery and for building state and local capacities may overcome current challenges in vector-borne disease prevention and control, but will require coordination across a national network of collaborators operating under a national strategy. Such an effort should reduce the impact of emerging vectors and could reverse the increasing trend of vector-borne disease incidence and associated morbidity and mortality.

Mosquito-borne and tick-borne disease incidence is increasing in the United States, with a tripling of reported cases annually from 27,388 cases in 2004 to 96,075 cases in 2016.^1^ Two distinct trends account for this increase: steadily increasing tick-borne disease incidence and interspersed, sporadic outbreaks caused by an expanding array of domestic and invasive mosquito-borne pathogens. Acceleration of many underlying causes of these trends, such as expanding travel and trade, urbanization, changing land use, increasing temperatures, and population growth, portends that public health and health-care delivery systems will continue to face an expanding spectrum of vector-borne pathogens of increasing incidence and distribution.^2,3^

Effective response to vector-borne diseases depends on robust public health surveillance and laboratory systems to detect new pathogens and emerging trends, proven prevention and mitigation measures, scalable capacity to implement these measures, sensitive and specific diagnostics, and effective therapeutics. Although federal, state, and local efforts following the introduction of West Nile virus into New York City, NY in 1999 bolstered many of these capacities, progress has been uneven and sporadic, leaving considerable gaps in our response architecture. Further complicating the response is vector-borne disease transmission across the U.S.–Mexico and U.S.–Canada borders.

The creation of the ArboNET surveillance system in 2000 following the introduction of West Nile virus in the United States enabled timely arboviral disease monitoring nationwide for the first time. ArboNET monitors 25 arboviral diseases, including nationwide Zika virus surveillance. Although ArboNET uniquely has capacity to simultaneously monitor viral activity in mosquitoes, animals, and humans, decreased support has eroded its capabilities over time.^4^ The bacterial, rickettsial, and parasitic vector-borne diseases are tracked through the National Notifiable Disease Surveillance System; many of these diseases are underreported. For example, the sheer number of Lyme disease cases, now estimated at more than 300,000 annually, has stressed surveillance systems and led to considerable underreporting in some areas.^5,6^ These limitations limit our ability to monitor and control the geographic expansion of disease vectors in the United States.

Proven and scalable control measures do not exist for many vector-borne diseases. Particularly concerning is the lack of environmental and entomological control measures for the *Ixodes scapularis* tick, the vector of at least seven human pathogens including those responsible for causing Lyme disease, anaplasmosis, and babesiosis.^7–13^ As a result, transmission of these pathogens continues largely unabated over expanding geographic areas. Another concern is the *Aedes aegypti* mosquito, which transmits the Zika, chikungunya, yellow fever, and dengue viruses. Whereas elimination of aquatic habitats and the application of dichlorodiphenyltrichloroethane enabled eradication of this mosquito from many countries in the middle of the last century, control measures in modern urban contexts have been largely ineffectual.^14^

Even when proven entomological control measures do exist, implementation may not be timely, may not be effective, or may not occur at all. Community vector control in the United States is conducted by a patchwork of vector-control operations whose capabilities vary widely. A recent survey of 1,083 vector-control organizations indicated that 84% lacked at least one of five core capacities deemed essential for effective vector-control operations.^15^ Of particular concern, infrequent monitoring of insecticide resistance has likely contributed to inappropriate use of insecticides and resulted in high-level resistance in many areas.

Given these limitations, personal protection may be the only viable option in many situations; however, personal protection measures have no impact on enzootic transmission and compliance is often low.^16^ Daily tick checks and prompt removal of attached ticks can reduce the risk of becoming infected with Lyme disease bacteria because the risk of transmission from an infected tick increases with every day the tick is allowed to remain attached^17^; however, other tick-borne pathogens such as Powassan virus and *Borrelia hermsii*, a causative agent of tick-borne relapsing fever, can be transmitted soon after tick bite. For example, P. virus may be transmitted in as little as 15 minutes after tick bite.^17^ No licensed vaccine exists for any domestic vector-borne disease pathogen.

Effective health-care delivery depends on early and accurate diagnosis and treatment. However, improvements in diagnostic testing platforms are required. For example, sensitive diagnostic tests are lacking for early-stage Lyme disease and test results from complicated diagnostic algorithms for its later stages are easily misinterpreted. Similarly, diagnostic tests are insensitive in early Rocky Mountain spotted fever, a disease of extremely high morbidity and mortality when treatment is delayed. The cross-reactivity of antibody tests among the flaviviruses continues to challenge serologic diagnosis of Zika virus in dengue-endemic areas. No proven therapeutic is available for arboviral diseases.

Without a concerted and sustained effort to address these deficiencies, little hope exists to reverse the upward trend of vector-borne disease incidence, morbidity, and mortality. Innovation based on a solid basic science foundation is required to improve diagnostics, to develop prevention and control methods proven to reduce human disease incidence, and to develop and test new therapeutics and vaccines. Translating this science into action will require strengthened public health systems at all levels, including improving capacities of vector-control operations. Harmonization of methods, when appropriate, will help to ensure operational consistency.

Innovation and discovery for vector-borne diseases face formidable challenges, including those resulting from the complex interplay between vectors, animal reservoirs, and humans. Interventions that appear promising under laboratory conditions may prove ineffective in the field, and those effective in reducing vector populations in the field may not reduce human disease incidence.^18^ Thus, large-scale field trials with both entomologic and human outcomes are required to validate new interventions. However, entomological efforts have recently shifted toward high technology and basic science laboratory research, creating a dearth of trained medical entomologists who can guide field operational research programs and respond to emergencies. For example, the Centers for Disease Control and Prevention (CDC) only employed a dozen medical entomologists when the Zika virus epidemic began, a particularly striking shortage considering CDC’s original mission to combat vector-borne diseases.^19^ With the current workforce and effort, new and proven options for vector control are unlikely to become available in the near future.

Despite the aforementioned limitations, building and sustaining state and local capacities hold considerable promise for reducing disease incidence, morbidity, and mortality. For example, large-scale, multipronged public health efforts reduced tick burden to near zero, resulting in a striking reduction in the incidence of Rocky Mountain spotted fever in select tribal communities in Arizona.^20^ Sustained local mosquito surveillance can detect impending West Nile virus outbreaks even before human cases are recorded, enabling timely prevention efforts.^21,22^ Insecticide resistance monitoring and management can help ensure that insecticides will be effective when mosquito-borne outbreaks occur. Robust laboratory capacity promotes timely diagnosis and treatment. Health promotion efforts can help to increase compliance with personal protection efforts.

To expand public health impact, CDC has invested in workforce development, innovation and discovery, and state capacity building. Specifically, five university-based, vector-borne disease centers of excellence have been funded to 1) train the next generation of medical entomologists, 2) establish communities of practice, and 3) conduct applied research, such as ways to monitor and reduce insecticide resistance. These centers were funded with Zika supplemental funds and are funded through 2021; future funding of such vector-borne disease centers of excellence will depend on the identification of a future source of funding. In addition, a network of professional, nongovernmental organizations are funded to assess and monitor local and state capacities and build capacity of front-line vector-control workers. Partnerships now exist with industry and academia to develop and test new vector-control tools, such as novel public health pesticides and repellents, and to develop improved diagnostics. CDC participates in the intergovernmental response in areas of shared responsibilities, including the development of vaccines, therapeutics, and diagnostics. State and territorial health departments receive funding to improve local and state capacities for surveillance, laboratory diagnostics, and prevention. In addition to core funding to all states and territories to maintain basic surveillance and laboratory capacities, CDC plans to expand funding to high-risk states through a competitive process, as funds permit, to develop model vector-borne disease prevention and control programs.

Taken together, addressing the dual needs for innovation and discovery and for building state and local capacities will require a national network of collaborators. CDC has engaged federal agencies, health departments, academic partners, public health partners, innovators from academia and industry, and the community (Figure 1).

**Figure 1.**
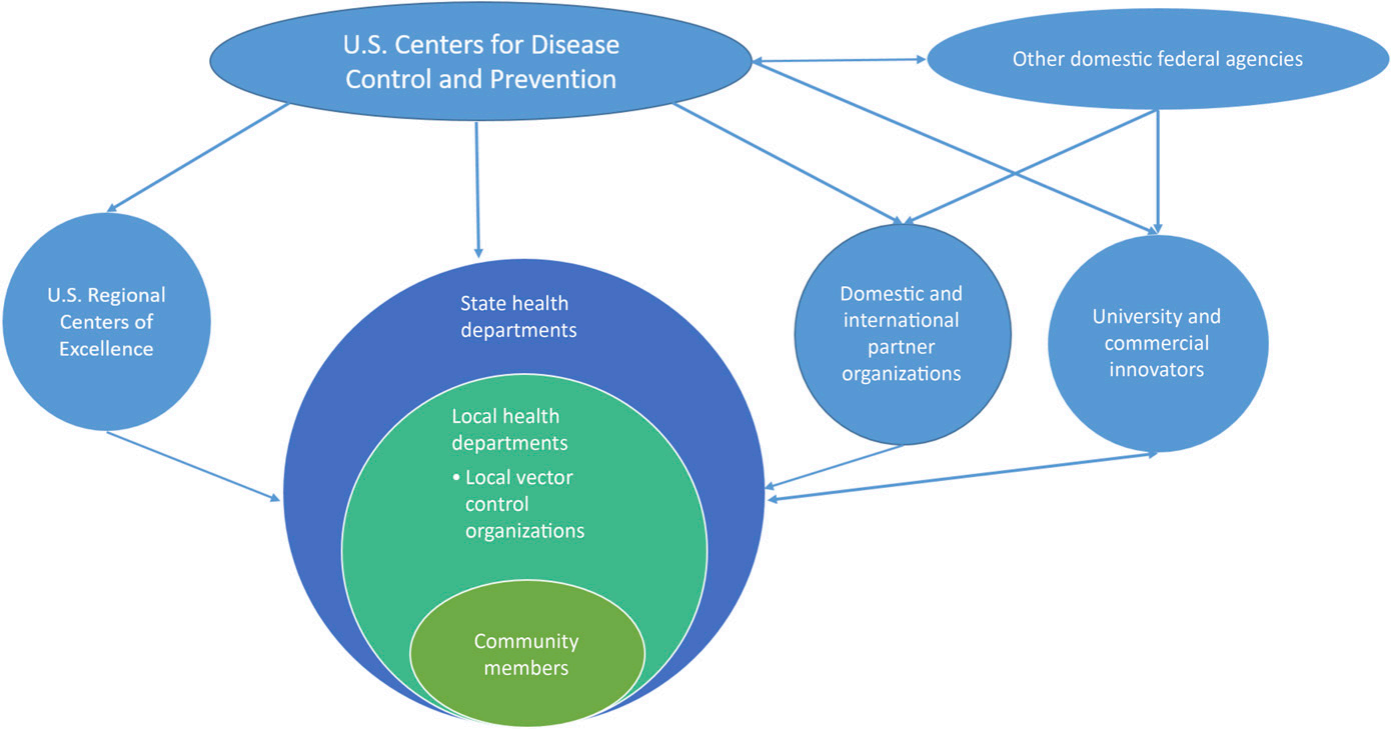
Domestic vector-borne disease prevention and control system map. This figure appears in color at www.ajtmh.org.

The need to activate a vector-borne disease prevention and control network is exemplified by the recent domestic emergence of *Haemaphysalis longicornis*, also known as the Asian longhorned tick. The last known introduction of an invasive tick is thought to be approximately 80 years ago.^23^ *H. longicornis* was first brought to the attention of the Hunterdon County Health Department on August 1, 2017, by a resident who found this invasive tick on a domesticated sheep.^24^ This tick is a vector of many vector-borne pathogens in Asia, including severe fever with thrombocytopenia syndrome virus in Asia.^25^ Severe fever with thrombocytopenia syndrome virus is closely related to Heartland virus, a pathogenic tick-borne virus found in the Southeastern United States.^26,27^ *H. longicornis* has been identified subsequently in an expanding number of states (Arkansas, Connecticut, Maryland, New York, North Carolina, Pennsylvania, Virginia, and West Virginia as of September 2018), raising the possibility of its unrecognized presence for some time.^28^ To date, no human cases of disease have been associated with this tick in the United States; however, based on the experience in Asia, the possibility is clear. Research to understand its distribution and potential to transmit pathogens found in North America, to determine whether human illnesses are occurring following *H. longicornis* bites, and to identify safe and effective vector-control strategies will require a skilled entomologic, microbiologic, ecologic, and epidemiologic public health workforce at federal, state, and local levels working in concert with academic partners.

The solutions required to build national resilience for the vector-borne diseases are complex and not easily forthcoming. However, the ongoing threat of vector-borne disease has become increasingly obvious, with the emergence of *H. longicornis* serving as the most recent example of an invasive vector arriving on our shores. Although success in all arenas cannot be guaranteed, a concerted, sustained national effort is needed among a network of partners operating under a national strategy to address the dual needs of *improved innovation and discovery* and *building state and local capacities*. Such an effort should reduce the impact of emerging vectors and could reverse the increasing trend of vector-borne disease incidence and associated morbidity and mortality. At a minimum, a national vector-borne disease prevention and control strategy should include strategic priorities that seek to improve national vector and human case surveillance; vector-borne disease prevention, diagnosis, and treatment efforts; and state and local capacities to implement vector-borne disease prevention and control programs.
